# Hypoxia-induced treatment failure in advanced squamous cell carcinoma of the uterine cervix is primarily due to hypoxia-induced radiation resistance rather than hypoxia-induced metastasis

**DOI:** 10.1054/bjoc.2000.1266

**Published:** 2000-07-03

**Authors:** E K Rofstad, K Sundfør, H Lyng, C G Tropé

**Affiliations:** Departments of Biophysics, Gynecology, Institute for Cancer Research, The Norwegian Radium Hospital, Montebello, Oslo, N-0310, Norway

**Keywords:** cervix carcinoma, hypoxia, oxygen tension, metastasis, radiation resistance

## Abstract

Poor outcome of treatment in advanced cervix carcinoma has been shown to be associated with poor oxygenation of the primary tumour. Hypoxia may cause radiation resistance and promote lymph-node metastasis. The purpose of the study reported here was to investigate whether hypoxia-induced treatment failure in advanced cervix carcinoma is primarily a result of hypoxia-induced radiation resistance or the presence of hypoxia-induced lymph-node metastases at the start of treatment. Thirty-two patients with squamous cell carcinoma of the uterine cervix were included in the study. Radiation therapy was given with curative intent as combined external irradiation and endocavitary brachytherapy. The oxygenation status of the primary tumour was measured prior to treatment using the Eppendorf *p* O _2_ Histograph. Pelvic and para-aortal lymph-node metastases were detected by magnetic resonance imaging at the time of initial diagnosis. The primary tumours of the patients with metastases (*n* = 18) were significantly more poorly oxygenated than those of the patients without metastases (*n* = 14). Multivariate Cox regression analyses involving biological and clinical parameters identified the tumour subvolume having *p* O _2_ values below 5mmHg (HSV (*p* O _2_< 5mmHg) as the only significant, independent prognostic factor for locoregional control, disease-free survival and overall survival. The probabillities of locoregional control, disease-free survival and overall survival were significantly lower for the patients with HSV (*p* O _2_< 5 mmHg) above the median value than for those with HSV (*p* O _2_< 5 mmHg) below the median value. On the other hand, the outcome of treatment was not significantly different for the patients with metastases and the patients without metastases at the start of treatment, irrespective of clinical end-point. Consequently, treatment failure was primarily a result of hypoxia-induced radiation resistance rather than hypoxia-induced lymph-node metastasis, suggesting that novel treatment strategies aiming at improving tumour oxygenation or enhancing the radiation sensitivity of hypoxic tumour cells may prove beneficial in attempts to improve the radiation therapy of advanced cervix carcinoma. © 2000 Cancer Research Campaign


*British Journal of Cancer* (2000) **83**(3), 354–359
© 2000 Cancer Research Campaign
doi: 10.1054/ bjoc.2000.1266, available online at http://www.idealibrary.com on IDEAL®

### Hypoxia-induced treatment failure in advanced squamous cell carcinoma of the uterine cervix is primarily due to hypoxia-induced radiation resistance rather than hypoxia-induced metastasis

**EK Rofstad[1], K Sundfør[2], H Lyng[1] and CG Tropé[2]**

Departments of [1]Biophysics and [2]Gynecology, Institute for Cancer Research, The Norwegian Radium Hospital, Montebello, N-0310 Oslo, Norway

**Summary** Poor outcome of treatment in advanced cervix carcinoma has been shown to be associated with poor oxygenation of the primary tumour. Hypoxia may cause radiation resistance and promote lymph-node metastasis. The purpose of the study reported here was to investigate whether hypoxia-induced treatment failure in advanced cervix carcinoma is primarily a result of hypoxia-induced radiation resistance or the presence of hypoxia-induced lymph-node metastases at the start of treatment. Thirty-two patients with squamous cell carcinoma of the uterine cervix were included in the study. Radiation therapy was given with curative intent as combined external irradiation and endocavitary brachytherapy. The oxygenation status of the primary tumour was measured prior to treatment using the Eppendorf $pO_2$ Histograph. Pelvic and para-aortal lymph-node metastases were detected by magnetic resonance imaging at the time of initial diagnosis. The primary tumours of the patients with metastases ($n$ = 18) were significantly more poorly oxygenated than those of the patients without metastases ($n$ = 14). Multivariate Cox regression analyses involving biological and clinical parameters identified the tumour subvolume having $pO_2$ values below 5mmHg (HSV ($pO_2$ < 5mmHg) as the only significant, independent prognostic factor for locoregional control, disease-free survival and overall survival. The probabillities of locoregional control, disease-free survival and overall survival were significantly lower for the patients with HSV ($pO_2$ < 5 mmHg) above the median value than for those with HSV ($pO_2$ < 5 mmHg) below the median value. On the other hand, the outcome of treatment was not significantly different for the patients with metastases and the patients without metastases at the start of treatment, irrespective of clinical end-point. Consequently, treatment failure was primarily a result of hypoxia-induced radiation resistance rather than hypoxia-induced lymph-node metastasis, suggesting that novel treatment strategies aiming at improving tumour oxygenation or enhancing the radiation sensitivity of hypoxic tumour cells may prove beneficial in attempts to improve the radiation therapy of advanced cervix carcinoma. © 2000 Cancer Research Campaign

**Keywords**: cervix carcinoma; hypoxia; oxygen tension; metastasis; radiation resistance

Squamous cell carcinomas of the uterine cervix frequently develop hypoxic regions during growth (Höckel et al, 1991; Brizel et al, 1995; Wong et al, 1997; Cooper et al, 1999; Dunst et al, 1999). Hypoxia may cause resistance to radiation therapy (Stone et al, 1993) and increased metastatic propensity (Hill, 1990). Several studies of advanced cervix carcinoma have demonstrated a positive association between poor outcome of radiation therapy and poor oxygenation of the primary tumour, measured with the Eppendorf $pO_2$ histograph prior to treatment (Höckel et al, 1993; 1996; Fyles et al, 1998*a*; 1998*b*). The worse treatment outcome of the patients with the most hypoxic tumours could be related to the classical oxygen effect (Bush, 1986; Overgaard et al, 1989) or to the development of metastases associated with hypoxia (Höckel et al, 1996; Sundfør et al, 1998).

Identification of the primary cause of treatment failure in advanced cervix carcinoma may facilitate the development of improved treatment strategies for this disease. Thus, if treatment failure is primarily a result of hypoxia-induced radiation resistance, treatment strategies aiming at improving tumour oxygenation or enhancing the radiation sensitivity of hypoxic cells may prove beneficial. On the other hand, if treatment failure is primarily a result of the presence of hypoxia-induced metastases at the start of treatment, other strategies are required to improve the outcome of treatment.

Studies at The Norwegian Radium Hospital have confirmed that poor outcome of treatment of advanced cervix carcinoma is associated with poor oxygenation of the primary tumour; tumour hypoxia was identified as an important prognostic factor for locoregional control, disease-free survival and overall survival (Sundfør et al, 2000). In the present work, the outcome of radiation therapy of advanced cervix carcinoma was investigated in relation to pretreatment oxygenation and metastatic status, in an attempt to reveal whether treatment failure is primarily a consequence of hypoxia-induced radiation resistance or hypoxia-induced metastasis.

### MATERIALS AND METHODS

### Patients and treatment

Thirty-two consecutive patients with squamous cell carcinoma of the uterine cervix were included in the study. The inclusion criteria required that: (a) the largest diameter of the primary

*Received 30 November 1999*
*Revised 3 April 2000*
*Accepted 6 April 2000*

*Correspondence to:* EK Rofstad

**Table 1** Tumour characteristics and outcome of treatment

| Patient | FIGO stage | Volume ($cm^3$) | Pathological lymph nodes (*n*) | Median $pO_2$ (mmHg) | HF ($pO_2$ < 5 mmHg) (%) | HSV ($pO_2$ < 5 mmHg) ($cm^3$) | Locoregional control Time (mo)[a] | Locoregional control Status[b] | Disease-free survival Time (mo)[a] | Disease-free survival Status[c] | Overall survival Time (mo)[a] | Overall survival Status[d] |
|---|---|---|---|---|---|---|---|---|---|---|---|---|
| 1 | 3B | 104 | 1 | 3.2 | 92 | 96 | 9 | 0 | 9 | 1 | 30 | 1 |
| 2 | 2B | 16 | 0 | 1.0 | 69 | 11 | 68 | 0 | 68 | 0 | 68 | 0 |
| 3 | 2B | 41 | 0 | 25.3 | 8 | 3 | 68 | 0 | 68 | 0 | 68 | 0 |
| 4 | 3B | 105 | 0 | 8.6 | 36 | 37 | 67 | 0 | 67 | 0 | 67 | 0 |
| 5 | 3B | 204 | 8 | 1.2 | 70 | 143 | 4 | 1 | 4 | 1 | 8 | 1 |
| 6 | 2B | 53 | 0 | 2.3 | 71 | 38 | 6 | 1 | 6 | 1 | 37 | 1 |
| 7 | 2B | 41 | 0 | 2.4 | 82 | 34 | 38 | 0 | 38 | 1 | 65 | 0 |
| 8 | 2B | 37 | 1 | 2.1 | 69 | 25 | 64 | 0 | 64 | 0 | 64 | 0 |
| 9 | 2B | 73 | 0 | 2.3 | 78 | 57 | 63 | 0 | 63 | 0 | 63 | 0 |
| 10 | 4A | 88 | 0 | 1.6 | 77 | 68 | 16 | 1 | 16 | 1 | 25 | 1 |
| 11 | 3B | 62 | 2 | 11.4 | 46 | 29 | 62 | 0 | 62 | 0 | 62 | 0 |
| 12 | 3B | 151 | 2 | 1.2 | 74 | 112 | 7 | 0 | 7 | 1 | 14 | 1 |
| 13 | 2B | 139 | 0 | 6.3 | 44 | 62 | 4 | 1 | 4 | 1 | 22 | 1 |
| 14 | 4A | 185 | 4 | 1.1 | 93 | 171 | 4 | 0 | 4 | 1 | 9 | 1 |
| 15 | 3B | 41 | 0 | 1.2 | 82 | 34 | 23 | 1 | 23 | 1 | 24 | 1 |
| 16 | 2B | 30 | 3 | 6.8 | 28 | 8 | 51 | 0 | 51 | 0 | 51 | 0 |
| 17 | 2B | 59 | 1 | 2.5 | 73 | 43 | 36 | 1 | 36 | 1 | 49 | 0 |
| 18 | 3B | 138 | 4 | 0.9 | 76 | 105 | 17 | 0 | 17 | 1 | 50 | 0 |
| 19 | 2B | 141 | 1 | 0.9 | 94 | 132 | 12 | 1 | 12 | 1 | 24 | 1 |
| 20 | 2A | 27 | 0 | 0.9 | 73 | 20 | 49 | 0 | 49 | 0 | 49 | 0 |
| 21 | 2B | 21 | 0 | 6.5 | 47 | 10 | 45 | 0 | 45 | 0 | 45 | 0 |
| 22 | 2B | 56 | 0 | 10.0 | 11 | 6 | 13 | 0 | 13 | 1 | 25 | 1 |
| 23 | 2B | 22 | 3 | 1.9 | 81 | 18 | 43 | 0 | 43 | 0 | 43 | 0 |
| 24 | 2B | 43 | 3 | 2.6 | 61 | 27 | 41 | 0 | 41 | 0 | 41 | 0 |
| 25 | 2B | 212 | 8 | 1.3 | 87 | 185 | 4 | 0 | 4 | 1 | 34 | 1 |
| 26 | 2B | 35 | 0 | 24.4 | 16 | 6 | 39 | 0 | 39 | 0 | 39 | 0 |
| 27 | 1B | 166 | 2 | 4.4 | 54 | 90 | 37 | 0 | 37 | 0 | 37 | 0 |
| 28 | 2B | 55 | 1 | 3.0 | 87 | 48 | 12 | 0 | 12 | 1 | 15 | 1 |
| 29 | 2B | 99 | 2 | 1.6 | 95 | 94 | 33 | 0 | 33 | 0 | 33 | 0 |
| 30 | 2B | 69 | 3 | 1.5 | 83 | 57 | 31 | 0 | 31 | 0 | 31 | 0 |
| 31 | 2B | 100 | 2 | 3.9 | 64 | 64 | 12 | 0 | 12 | 1 | 21 | 1 |
| 32 | 1B | 7 | 0 | 1.2 | 71 | 5 | 31 | 0 | 31 | 0 | 31 | 0 |

[a]Time (mo), observation time in months. [b]Status, patient with locoregional control 0; patient without locoregional control 1. [c]Status, patient alive and disease-free 0; patient dead or alive and not disease-free 1. [d]Status, patient alive 0; patient dead 1.

tumour, determined from pretreatment magnetic resonance (MR) images, was 2 cm or more; (b) the patients were less than 70 years of age; (c) the patients met the criteria for anaesthesia function class ASA I or ASA II; and (d) the patients were subjected to radiation therapy without surgery of the primary tumour. Important characteristics of the patients are listed in Table 1. Clinical stage was determined according to the FIGO criteria. Pretreatment tumour volume (*V*) was calculated as $V = \pi/6 \cdot a \cdot b \cdot c$, where *a*, *b* and *c* are three orthogonal diameters determined from MR images. The study was approved by the local ethical commitee, and informed consent was achieved from the patients.

The metastatic status of the patients was assessed at the time of initial diagnosis. Pelvic and para-aortal lymph-node metastases were detected by MR imaging. Lymph nodes were considered pathological when the minimal axial diameter was 10 mm or more or when the minimal axial diameter was 8–10 mm and the lymph nodes were judged to be round. The sensitivity, specificity, accuracy and positive predictive value of these criteria in assessment of the metastatic status of lymph nodes have been shown to be high (Jager et al, 1996).

Radiation therapy was given with curative intent as combined external irradiation and endocavitary brachytherapy. The external irradiation was delivered with a 10 or 16 MV linear accelerator. The total dose was 50 Gy, given to the pelvic region with a four-field box technique in daily fractions of 2 Gy five times a week. The brachytherapy was delivered with a high dose-rate $^{192}$Ir after-loading machine. The total dose was 29–34 Gy, given to point A in 7–8 fractions. The follow-up included clinical examinations every third month for the first 2 years and thereafter every sixth month. MR of retroperitoneum and X-ray of thorax were performed during the first examination, after 2 months, after 1 year and when symptoms of recurrent disease were seen. Three end-points were used: overall survival, disease-free survival and locoregional control. Locoregional control was defined as complete and persistent tumour remission within the radiation field. The median follow-up, calculated from the start of treatment, was 50 months (range 31–69 months).

### Oxygen tension

Tumour oxygen tension ($pO_2$) was measured before treatment using a polarographic needle electrode (Eppendorf $pO_2$ Histograph 6650) (Sundfør et al, 1997). Heart rate, arterial blood pressure and arterial $HbO_2$ saturation were recorded throughout the $pO_2$ measurements. A total of 57–252 $pO_2$ readings in 2–6 tracks were performed in each tumour. The tracks were located peripherally (clock positions 3, 6, 9 and 12) or centrally and were directed perpendicularly to the tumours. The track lengths were determined by the tumour size, measured by analysing MR images. A $pO_2$ frequency distribution was generated for each tumour by pooling the data from the individual tracks. Three $pO_2$ parameters were derived from the tumours: median $pO_2$, the fraction of the $pO_2$ readings giving $pO_2$ values below 5 mmHg (HF ($pO_2$ < 5 mmHg)) and the tumour subvolume having $pO_2$ values below 5 mmHg (HSV ($pO_2$ < 5 mmHg)), where HSV ($pO_2$ < 5 mmHg) = HF ($pO_2$ < 5 mmHg) × *V* (tumour volume).

### Statistical analysis

Volume and oxygenation parameters of metastatic and non-metastatic tumours were compared by using the Student's *t*-test. Probabilities of locoregional control, disease-free survival or overall survival of two groups of patients were compared by actuarial analysis, using a log-rank test in Kaplan–Meier estimates. Univariate and multivariate Cox regression analyses of continuous or categorical data were used to evaluate the prognostic value of tumour parameters. The prognostic value of tumour stage was investigated by joining stage 1 and 2 in one group and stage 3 and 4 in a second group. A significance criterion of $P < 0.05$ was used.

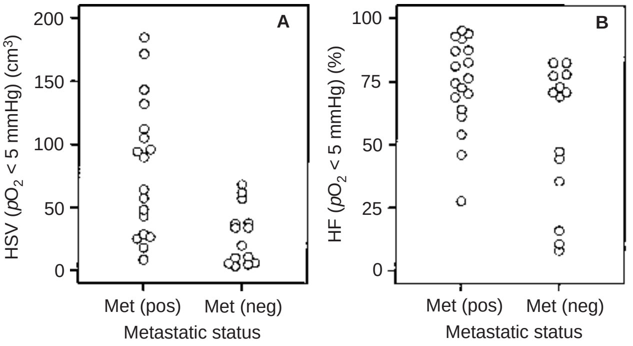

**Figure 1** (**A**) HSV ($pO_2$ < 5 mmHg) and (**B**) HF ($pO_2$ < 5 mmHg) of the primary tumour of 32 patients with advanced squamous cell carcinoma of the uterine cervix. Patients with metastases and patients without metastases at the time of $pO_2$ measurement are compared. Points represent individual patients.

### RESULTS

Tumour oxygenation status differed substantially among individual patients, although all tumours were highly heterogenous in $pO_2$. Median $pO_2$, HF ($pO_2$ < 5 mmHg) and HSV ($pO_2$ < 5 mmHg) of each individual patient are presented in Table 1 together with FIGO stage, tumour volume, number of pathological lymph nodes, observation time and outcome of treatment in terms of locoregional control, disease-free survival and overall survival.

Eighteen of the 32 patients had developed regional lymph-node metastases at the time of initial diagnosis. The number of pathological lymph nodes in these patients ranged from 1–8 (Table 1). Metastases could not be detected in the remaining 14 patients. Tumour oxygenation status differed substantially between the patients with metastases and the patients without metastases. The patients with metastases showed a significantly higher HSV ($pO_2$ < 5 mmHg) ($P = 0.002$) and a significantly higher HF ($pO_2$ < 5 mmHg) ($P = 0.03$) than the patients without metastases (Figure 1). On the other hand, median tumour $pO_2$ did not differ significantly between the two groups of patients.

HSV ($pO_2$ < 5 mmHg) was correlated to tumour volume ($P < 0.001$), as revealed by linear regression analysis. Tumour volume differed substantially between the patients with metastases and the patients without metastases as did HSV ($pO_2$ < 5 mmHg); the patients with metastases showed a significantly larger tumour volume ($P = 0.01$) than those without metastases (data not shown). The *P*-value of 0.002 pertaining to HSV ($pO_2$ < 5 mmHg) was substantially lower than that of 0.01 pertaining to tumour volume, suggesting that the discriminative power of HSV ($pO_2$ < 5 mmHg) was higher than that of tumour volume. There was no correlation between HF ($pO_2$ < 5 mmHg) and tumour volume ($P = 0.12$).

Univariate Cox regression analysis showed that disease-free survival was correlated to the number of pathological lymph nodes

 © 2000 Cancer Research Campaign

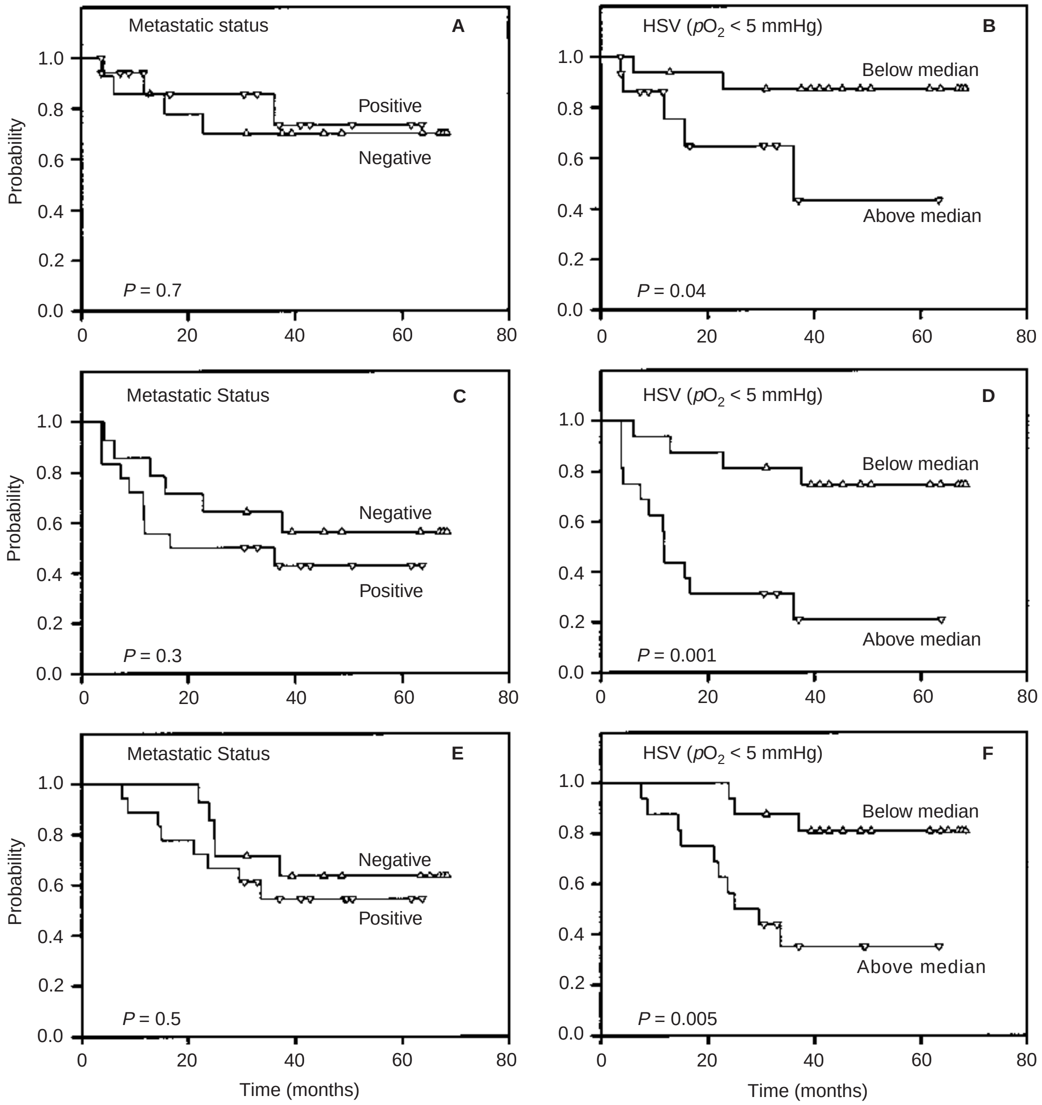

**Figure 2** Kaplan–Meier plots showing probability of (**A, B**) locoregional control, (**C, D**) disease-free survival and (**E, F**) overall survival for 32 patients with advanced squamous cell carcinoma of the uterine cervix. Patients with metastases and patients without metastasis at the start of radiation therapy are compared (**A, C, E**) and patients with HSV ($pO_2$ < 5 mmHg) above and HSV ($pO_2$ < 5 mmHg) below the median value are compared (**B, D, F**). Median HSV ($pO_2$ < 5 mmHg) was 40 $cm^3$. Censored observations are indicated as ▽ or △.

at the time of initial diagnosis, whereas locoregional control and overall survival were not (Table 2). Moreover, neither locoregional control, disease-free survival nor overall survival was correlated to metastatic status, i.e. the patients were catagorized as either metastasis positive or metastasis negative (Table 2). The probabilities of locoregional control, disease-free survival and overall survival were not significantly worse for the patients with metastases than for the patients without metastatic deposits at the start of treatment ($P$ = 0.7, $P$ = 0.3 and $P$ = 0.5, respectively), as illustrated by Kaplan–Meier plots in Figure 2.

Locoregional control, disease-free survival and overall survival showed significant correlations to HSV ($pO_2$ < 5 mmHg), as revealed by univariate Cox regression analysis (Table 2). In contrast, significant correlations between locoregional control, disease-free survival or overall survival on the one hand and HF ($pO_2$ < 5 mmHg) or median $pO_2$ on the other were not found (Table 2). The probabilities of locoregional control, disease-free survival and overall survival were significantly lower for the patients with HSV ($pO_2$ < 5 mmHg) above the median value than for those with HSV ($pO_2$ < 5 mmHg) below the median value

**Table 2** Results of univariate Cox regression analysis

| Parameter | *P*-value | | |
|---|---|---|---|
| | Locoregional control | Disease-free survival | Overall survival |
| Metastatic status (+/–) | 0.71 | 0.34 | 0.46 |
| Pathol. nodes (*n*) | 0.18 | 0.0068 | 0.086 |
| Median $pO_2$ | 0.28 | 0.14 | 0.23 |
| HF ($pO_2$ < 5 mmHg) | 0.22 | 0.056 | 0.14 |
| HSV ($pO_2$ < 5 mmHg) | 0.016 | <0.0001 | 0.0010 |
| FIGO stage | 0.18 | 0.043 | 0.040 |
| Tumour volume | 0.020 | 0.0001 | 0.0014 |

© 2000 Cancer Research Campaign

($P$ = 0.04, $P$ = 0.001 and $P$ = 0.005, respectively), as illustrated by Kaplan–Meier plots in Figure 2.

Univariate Cox regression analysis showed that locoregional control was correlated to tumour volume, and disease-free survival and overall survival were correlated to FIGO stage and tumour volume (Table 2). Moreover, multivariate Cox regression analysis including number of pathological lymph nodes, metastatic status, HSV ($pO_2$ < 5 mmHg), HF ($pO_2$ < 5 mmHg), FIGO stage and tumour volume identified HSV ($pO_2$ < 5 mmHg) as the only independent prognostic factor for locoregional control ($P$ = 0.006; relative risk per $cm^3$ = 1.04), disease-free survival ($P$ < 0.0001; relative risk per $cm^3$ = 1.03) and overall survival ($P$ = 0.001; relative risk per $cm^3$ = 1.02).

### DISCUSSION

The present study confirmed the results of previous studies suggesting that tumour oxygenation status is an important prognostic factor in advanced cervix carcinoma (Höckel et al, 1993; 1996; Fyles et al, 1998*a*; 1998*b*). Median $pO_2$ and HF ($pO_2$ < 5 mmHg) were used as parameters for tumour oxygenation status in these previous studies. In the present study, HSV ($pO_2$ < 5 mmHg) was found to have a higher prognostic value than median $pO_2$ or HF ($pO_2$ < 5 mmHg), irrespective of whether locoregional control, disease-free survival or overall survival was used as end-point. HSV ($pO_2$ < 5 mmHg) has not been recognized as a prognostic factor in advanced cervix carcinoma previously. However, our observation is consistent with the results from a similar study of squamous cell carcinoma of the head and neck (Stadler et al, 1999). In this study, oxygen tension was measured in primary tumours in 26 patients and in lymph-node metastases in 33 patients, and multivariate Cox regression analysis identified HSV ($pO_2$ < 5 mmHg) as a significant prognostic factor for overall survival.

The study reported here suggests that a high incidence of lymph-node metastases is associated with poor oxygenation of the primary tumour in advanced cervix carcinoma. Thus, the tumours of the patients with metastases at the time of initial diagnosis showed a higher HSV ($pO_2$ < 5 mmHg) and a higher HF ($pO_2$ < 5 mmHg) than those of the patients without metastases. This observation is consistent with data from a study of advanced cervix carcinoma by Höckel et al (1996) showing that tumours with a median $pO_2$ < 10 mmHg have significantly larger extensions, more frequent parametrial infiltration and more extensive lymph–vascular space involvement than tumours with a median $pO_2$ > 10 mmHg. Moreover, our data is also consistent with the observation that a high lactate concentration in the primary tumour is associated with a high incidence of metastases in advanced cervix carcinoma (Schwickert et al, 1995).

One possible interpretation of our observation is that tumour hypoxia may promote lymph node metastasis in cervix carcinoma. This interpretation is supported by results from studies of experimental tumours. Thus, exposure of tumour cells to hypoxia in vitro before intravenous inoculation in mice has been shown to increase the frequency of lung colonies in murine tumours (Young et al, 1988) and human melanoma xenografts (Rofstad and Danielsen, 1999). Several mechanisms have been identified that may be involved in hypoxia-induced metastasis of tumours (Rofstad, 2000). Thus, hypoxia may induce point mutations and DNA strand breakage leading to deletions, amplifications and genomic instability. Hypoxia may also provide a physiological pressure in tumours selecting for metastatic cell phenotypes. Moreover, hypoxia may induce a temporary increase in the expression of gene products involved in the metastatic cascade, either through gene amplifications or through normal physiological processes by activating oxygen sensors, hypoxia signal transduction pathways and DNA transcription factors (Rofstad, 2000).

The present study showed that the outcome of treatment was associated with FIGO stage, tumour volume and tumour oxygenation status prior to treatment, consistent with previous studies of advanced cervix carcinoma (Höckel et al, 1993; 1996; Fyles et al, 1998*a*; 1998*b*). In the study reported here, tumour oxygenation status, measured as HSV ($pO_2$ < 5 mmHg), showed a higher prognostic value than both FIGO stage and tumour volume. Thus, HSV ($pO_2$ < 5 mmHg) was found to be the only independent prognostic factor for locoregional control, disease-free survival and overall survival in multivariate Cox regression analyses involving a large number of biological and clinical parameters. Moreover, the probabilities of locoregional control, disease-free survival and overall survival were significantly lower for the patients with HSV ($pO_2$ < 5 mmHg) above the median value than for those with HSV ($pO_2$ < 5 mmHg) below the median value. These observations suggest that measurement of tumour oxygen tension with the Eppendorf $pO_2$ histograph prior to treatment may be a useful prognostic test in advanced cervix carcinoma.

Hypoxia-induced treatment failure was primarily a result of hypoxia-induced radiation resistance rather than the presence of hypoxia-induced metastases at the start of treatment. This conclusion follows from the Kaplan–Meier plots in Figure 2, which show that the outcome of treatment was significantly worse for the patients with HSV ($pO_2$ < 5 mmHg) above the median value than for those with HSV ($pO_2$ < 5 mmHg) below the median value, whereas the outcome of treatment was not significantly different for the patients with metastases and the patients without metastases at the start of treatment. Moreover, univariate Cox regression analyses revealed strong correlations between outcome of treatment and HSV ($pO_2$ < 5 mmHg), irrespective of clinical end-point, whereas a significant correlation between outcome of treatment and metastatic status was not found for any of the end-points. Finally, multivariate Cox regression analysis involving several biological and clinical parameters showed that HSV ($pO_2$ < 5 mmHg) was the only independent prognostic factor for outcome of treatment, irrespective of end-point. This does not mean that the outcome of treatment was not influenced by the presence of metastases at the start of treatment. In fact, a strong correlation was found between disease-free survival and number of pathological lymph nodes in univariate Cox regression analysis, suggesting that the presence of lymph-node metastases at the start of treatment indeed represents a therapeutic problem in advanced cervix carcinoma.

Novel strategies are required to improve the radiation therapy of advanced cervix carcinoma. The study reported here has significant implications for the choice of strategy. Our data suggest that treatment failure is primarily a consequence of the presence of hypoxic cells in the primary tumour during the radiation therapy rather than the presence of pathological lymph nodes at the start of treatment. Strategies aiming at improving the oxygenation of the primary tumour during the radiation therapy may therefore prove beneficial. Such strategies may involve the use of agents which improve the oxygen-carrying capacity of the blood, increase tumour blood-flow and/or decrease the respiration rate of the tumour cells (Vaupel, 1990; Horsman, 1995). Other potentially

© 2000 Cancer Research Campaign useful strategies include strategies aiming at killing or enhancing the radiation sensitivity of hypoxic cells in the primary tumour, possibly involving the use of hypoxia-selective cytotoxins (Brown and Giaccia, 1998) and/or hypoxic cell radiosensitizers (Coleman, 1988). This suggestion is consistent with the observation that patients with advanced cervix carcinoma with local treatment failure show a significantly higher incidence of metastatic disease following treatment and a significantly lower overall survival rate than those with local treatment control (Fagundes et al, 1992; Leibel and Fuks, 1992). It should be noticed, however, that improved locoregional control or overall survival could not be demonstrated in clinical trials of advanced cervix carcinoma investigating potential therapeutic benefits of nitroimidazole hypoxic cell radiosensitizers (Overgaard, 1994).

In conclusion, hypoxia in the primary tumour may promote lymph-node metastasis and cause radiation resistance in cervix carcinoma. Treatment failure in advanced cervix carcinoma is primarily a result of hypoxia-induced radiation resistance rather than the presence of hypoxia-induced lymph-node metastases at the start of treatment.

### ACKNOWLEDGEMENTS

Financial support was received from The Norwegian Cancer Society and The Bothner Foundation for Cancer Research.

### REFERENCES

Brizel DM, Rosner GL, Prosnitz LR and Dewhirst MW (1995) Patterns and variability of tumour oxygenation in human soft tissue sarcomas, cervical carcinomas, and lymph node metastases. *Int J Radiat Oncol Biol Phys* **32**: 1121–1125

Brown JM and Giaccia AJ (1998) The unique physiology of solid tumours: opportunities (and problems) for cancer therapy. *Cancer Res* **58**: 1408–1416

Bush RS (1986) The significance of anemia in clinical radiation therapy. *Int J Radiat Oncol Biol Phys* **12**: 2047–2050

Coleman CN (1988) Hypoxia in tumours: a paradigm for the approach to biochemical and physiologic heterogeneity. *J Natl Cancer Inst* **80**: 310–317

Cooper RA, West CML, Logue JP, Davidson SE, Miller A, Roberts S, Stratford IJ, Honess DJ and Hunter RD (1999) Changes in oxygenation during radiotherapy in carcinoma of the cervix. *Int J Radiat Oncol Biol Phys* **45**: 119–126

Dunst J, Hänsgen G, Lautenschläger C, Füchsel G and Becker A (1999) Oxygenation of cervical cancers during radiotherapy and radiotherapy + *cis*-retinoic acid/interferon. *Int J Radiat Oncol Biol Phys* **43**: 367–373

Fagundes H, Perez CA, Grigsby PW and Lockett MA (1992) Distant metastases after irradiation alone in carcinoma of the uterine cervix. *Int J Radiat Oncol Biol Phys* **24**: 197–204

Fyles AW, Milosevic M, Pintile M and Hill RP (1998*a*) Cervix cancer oxygenation measured following external radiation therapy. *Int J Radiat Oncol Biol Phys* **42**: 751–753

Fyles AW, Milosevic M, Wong R, Kavanagh M-C, Pintile M, Sun A, Chapman W, Levin W, Manchul L, Keane TJ and Hill RP (1998*b*) Oxygenation predicts radiation response and survival in patients with cervix cancer. *Radiother Oncol* **48**: 149–156

Hill RP (1990) Tumour progression: potential role of unstable genomic changes. *Cancer Metastasis Rev* **9**: 137–147

Höckel M, Schlenger K, Knoop C and Vaupel P (1991) Oxygenation of carcinomas of the uterine cervix: evaluation by computerized $O_2$ tension measurements. *Cancer Res* **51**: 6098–6102

Höckel M, Knoop C, Schlenger K, Vorndran B, Baussmann E, Mitze M, Knapstein PG and Vaupel P (1993) Intratumoural $pO_2$ predicts survival in advanced cancer of the uterine cervix. *Radiother Oncol* **26**: 45–50

Höckel M, Schlenger K, Aral B, Mitze M, Schäffer U and Vaupel P (1996) Association between tumour hypoxia and malignant progression in advanced cancer of the uterine cervix. *Cancer Res* **56**: 4509–4515

Horsman MR (1995) Nicotinamide and other benzamide analogs as agents for overcoming hypoxic cell radiation resistance in tumours. *Acta Oncol* **34**: 571–587

Jager GJ, Barentsz JO, Oosterhof GO, Witjes JA and Ruijs SJH (1996) Pelvic adenopathy in prostatic and urinary bladder carcinoma: MR imaging with a three-dimensional T1-weighted magnetization-prepared-rapid gradient-echo sequence. *Am J Roentgenol* **167**: 1503–1507

Leibel SA and Fuks Z (1992) Is local failure a cause of or a marker for metastatic dissemination in carcinoma of the uterine cervix? *Int J Radiat Oncol Biol Phys* **24**: 377–380

Overgaard J, Bentzen SM, Kolstad P, Kjørstad K, Davy M, Bertelsen K, Mäntylä M, Frankendal B, Skryten A, Lövquist I, Pedersen M, Sell A and Jensen RH (1989) Misonidazole combined with radiotherapy in the treatment of carcinoma of the uterine cervix. *Int J Radiat Oncol Biol Phys* **16**: 1069–1072

Overgaard J (1994) Clinical evaluation of nitroimidazoles as modifiers of hypoxia in solid tumours. *Oncol Res* **6**: 509–518

Rofstad EK (2000) Microenvironment-induced cancer metastasis. *Int J Radiat Biol* **76**: 589–605

Rofstad EK and Danielsen T (1999) Hypoxia-induced metastasis of human melanoma cells: involvement of vascular endothelial growth factor-mediated angiogenesis. *Br J Cancer* **80**: 1697–1707

Schwickert G, Walenta S, Sundfør K, Rofstad EK and Mueller-Klieser W (1995) Correlation of high lactate levels in human cervical cancer with incidence of metastasis. *Cancer Res* **55**: 4757–4759

Stadler P, Becker A, Feldmann HJ, Hänsgen G, Dunst J, Würschmidt F and Molls M (1999) Influence of the hypoxic subvolume on the survival of patients with head and neck cancer. *Int J Radiat Oncol Biol Phys* **44**: 749–754

Stone HB, Brown JM, Phillips TL and Sutherland RM (1993) Oxygen in human tumours: correlations between methods of measurement and response to therapy. *Radiat Res* **136**: 422–434

Sundfør K, Lyng H, Kongsgård U, Tropé C and Rofstad EK (1997) Polarographic measurement of $pO_2$ in cervix carcinoma. *Gynecol Oncol* **64**: 230–236

Sundfør K, Lyng H and Rofstad EK (1998) Tumour hypoxia and vascular density as predictors of metastasis in squamous cell carcinoma of the uterine cervix. *Br J Cancer* **78**: 822–827

Sundfør K, Lyng H, Tropé CG and Rofstad EK (2000) Treatment outcome in advanced squamous cell carcinoma of the uterine cervix: relationships to pretreatment tumour oxygenation and vascularization. *Radiother Oncol* **54**: 101–107

Vaupel P (1990) Oxygenation of human tumours. *Strahlenther Onkol* **166**: 377–386

Wong RKW, Fyles A, Milosevic M, Pintile M and Hill RP (1997) Heterogeneity of polarographic oxygen tension measurements in cervix cancer: an evaluation of within and between tumour variability, probe position, and track depth. *Int J Radiat Oncol Biol Phys* **39**: 405–412

Young SD, Marshall RS and Hill RP (1988) Hypoxia induces DNA overreplication and enhances metastatic potential of murine tumour cells. *Proc Natl Acad Sci USA* **85**: 9533–9537

*© 2000 Cancer Research Campaign*
